# Supplementary material for: Histone H2A variant H2A.B is enriched in transcriptionally active and replicating HSV-1 lytic chromatin
Source: J Virol. 2024 Mar 7;98(4):e02015-23. doi: 10.1128/jvi.02015-23 (PMC11019955; doi:10.1128/jvi.02015-23)
Supplement: Supplemental figures — Figures S1 and S2. [file jvi.02015-23-s0001.pdf]

Supplementary Material

Table S1

Figures S1, S2

Table S1. Summary information of the sequencing data

Figure S1. Endogenous histone H2B and flag tagged histone H2A, H2A.B, macroH2A, or H2B are homogeneously distributed throughout the HSV-1 genome regardless of the dynamic state of the HSV-1 chromatin.

A. Line graphs presenting the $\log_2$ HSV-1 coverage (read depth) plots at single nucleotide resolution from 50,000 HSV-1-aligned paired-end reads normalized by the IP coverage [(Flag IP/No IP) / (H2B IP/No IP)].

B-E. Line graphs presenting the $\log_2$ HSV-1 coverage plots from 50,000 HSV-1-aligned paired-end reads normalized IP coverage (Flag IP/No IP) or (H2B IP/ No IP) for cells expressing flag-tagged histone H2A **(B)**, H2A.B **(C)**, macroH2A **(D)**, or H2B **(E)**.

Sh, short accessible chromatin; Lo, long accessible chromatin; Pe, insoluble chromatin; ND NF, non-digested non-fractionated but sonicated chromatin; Gray rectangles, cartoon representation of the HSV-1 genome.

Figure S2. Flag tagged histones are equally incorporated into chromatin along the entire HSV-1 genome regardless of whether the entire genome is transcribed, or transcription is restricted to the IE loci, and no H2A variant is enriched or depleted from the IE loci when transcription is restricted to the IE genes only.

A. Line graphs presenting the log_2 HSV-1 coverage plots from 50,000 HSV-1-aligned paired-end reads normalized by the IP coverage [(Flag IP/No IP) / (H2B IP/No IP)]

B. Genome coverage of the normalized signal from the co-immunoprecipitation of each flag tagged histone divided by the normalized H2B signal in infections treated or not with CHX.

Un, untreated infection; CHX, cycloheximide-treated infection; Gray rectangles, cartoon representation of the HSV-1 genome.

Figure S1. A

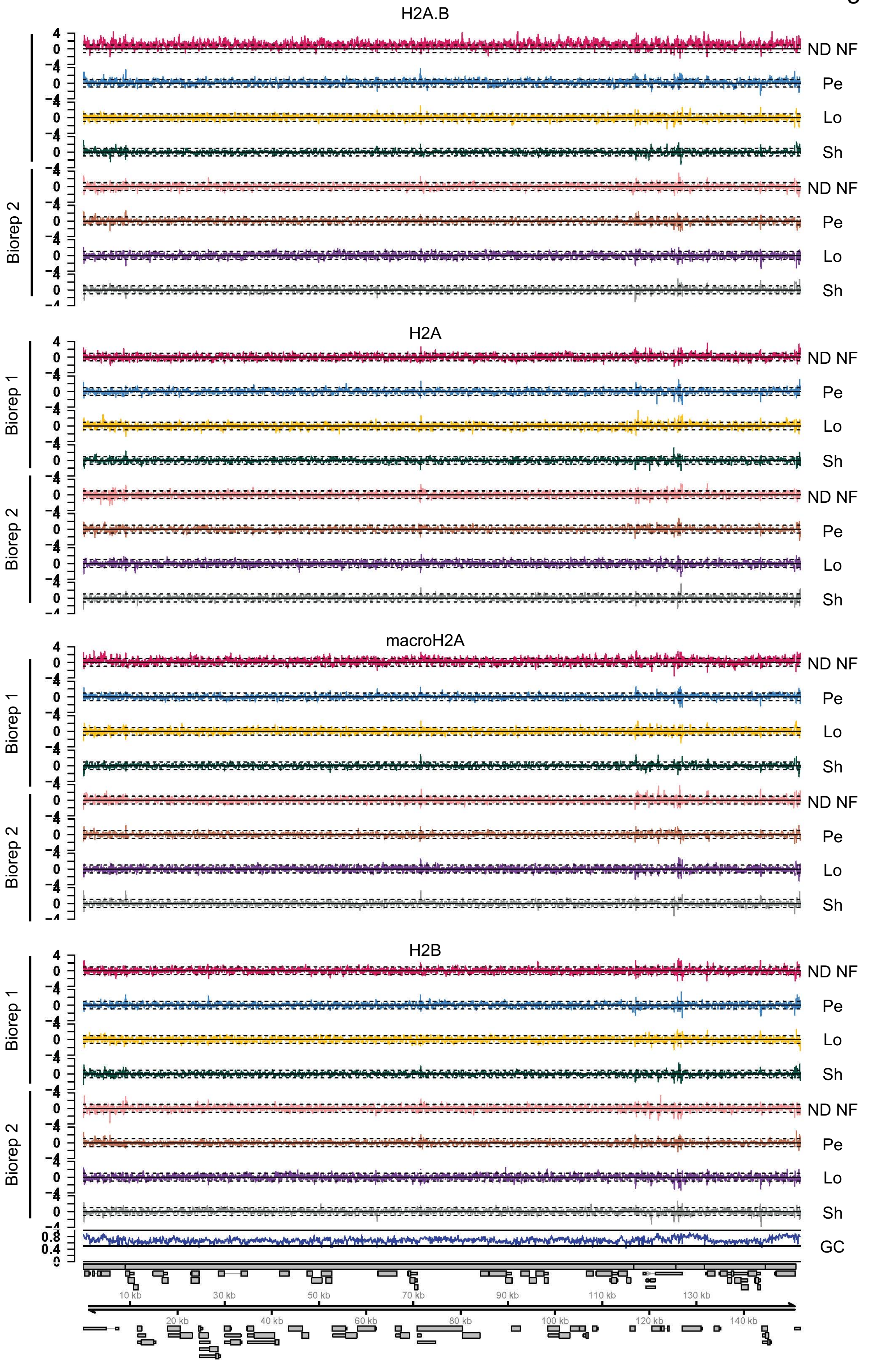

Figure S1. Endogenous histone H2B and flag tagged histone H2A, H2A.B, macroH2A, or H2B are homogeneously distributed throughout the HSV-1 genome regardless of the dynamic state of the HSV-1 chromatin.

Figure S1. B

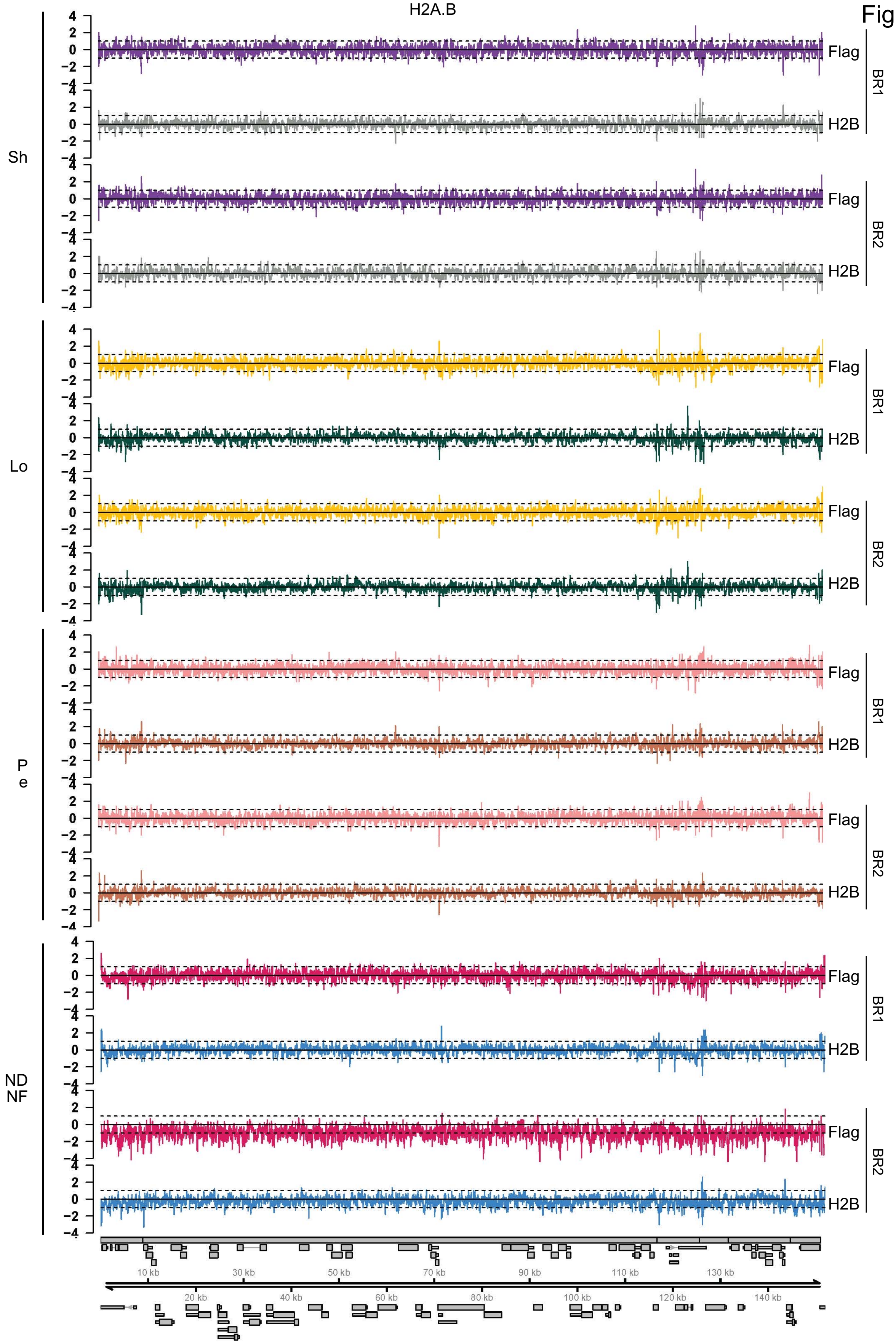

Figure S1. Endogenous histone H2B and flag tagged histone H2A, H2A.B, macroH2A, or H2B are homogeneously distributed throughout the HSV-1 genome regardless of the dynamic state of the HSV-1 chromatin.

Figure S1. C

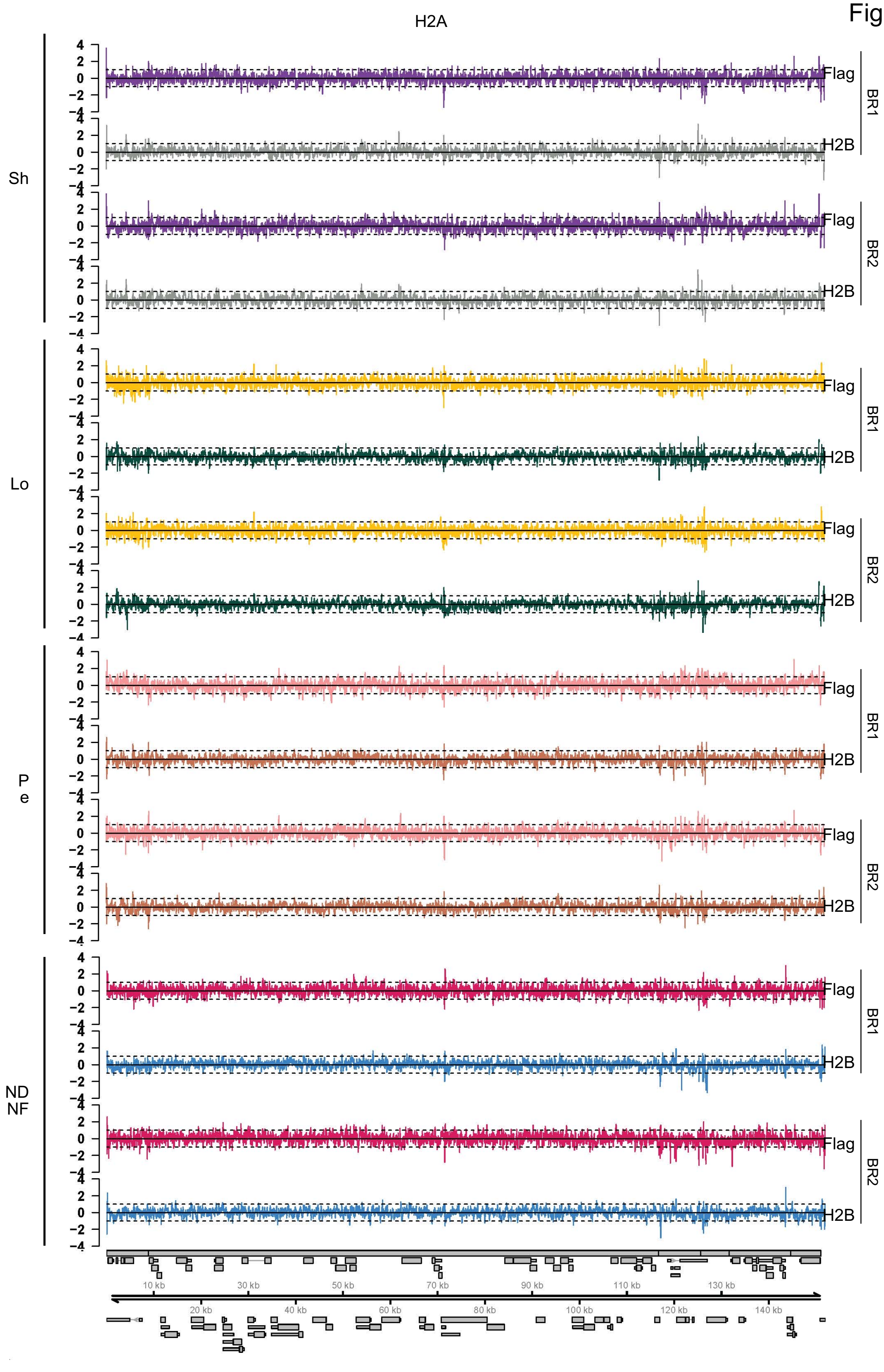

Figure S1. Endogenous histone H2B and flag tagged histone H2A, H2A.B, macroH2A, or H2B are homogeneously distributed throughout the HSV-1 genome regardless of the dynamic state of the HSV-1 chromatin.

Figure S1. D

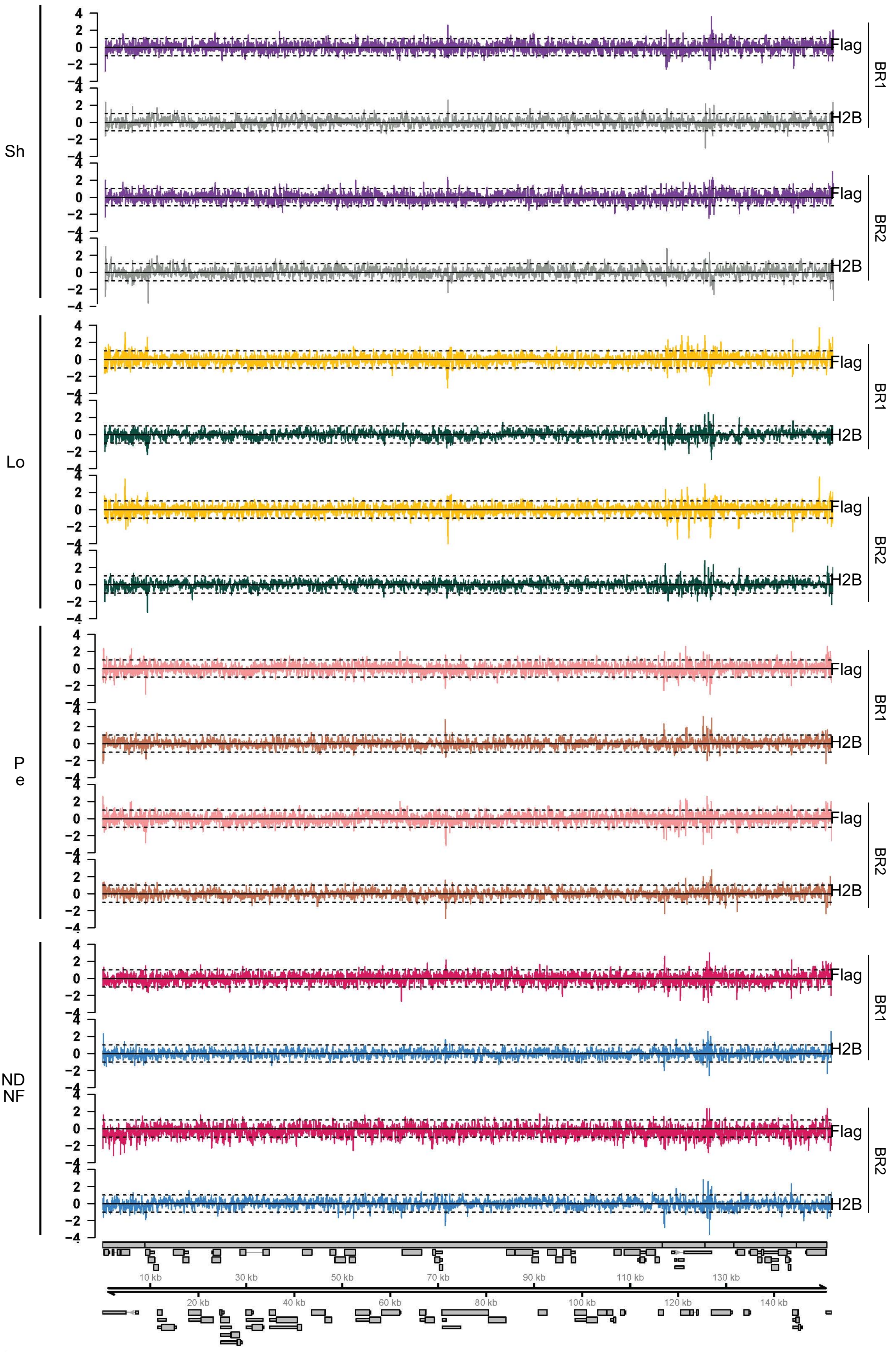

Figure S1. Endogenous histone H2B and flag tagged histone H2A, H2A.B, macroH2A, or H2B are homogeneously distributed throughout the HSV-1 genome regardless of the dynamic state of the HSV-1 chromatin.

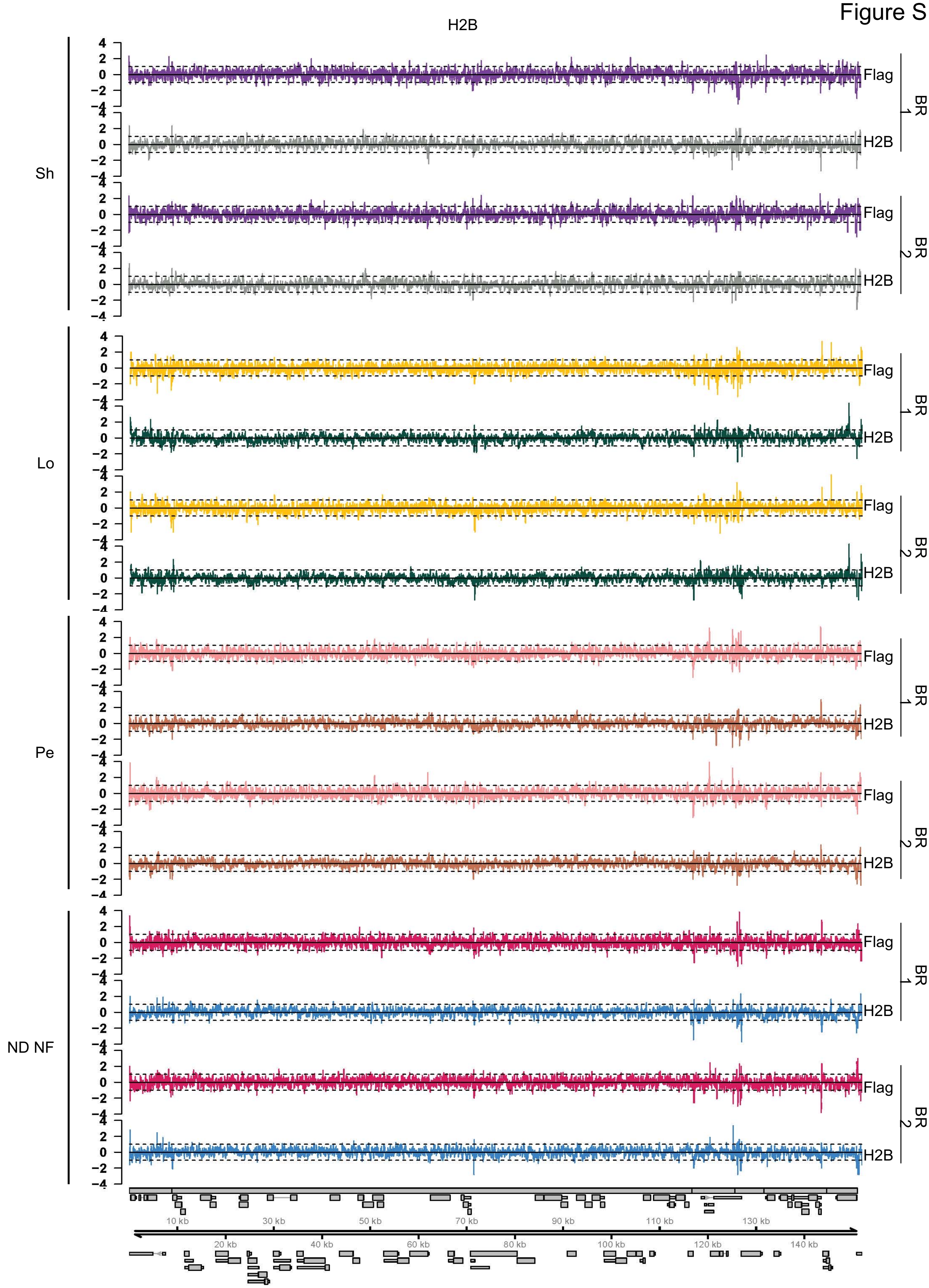

Figure S1. Endogenous histone H2B and flag tagged histone H2A, H2A.B, macroH2A, or H2B are homogeneously distributed throughout the HSV-1 genome regardless of the dynamic state of the HSV-1 chromatin.

Figure S2.A

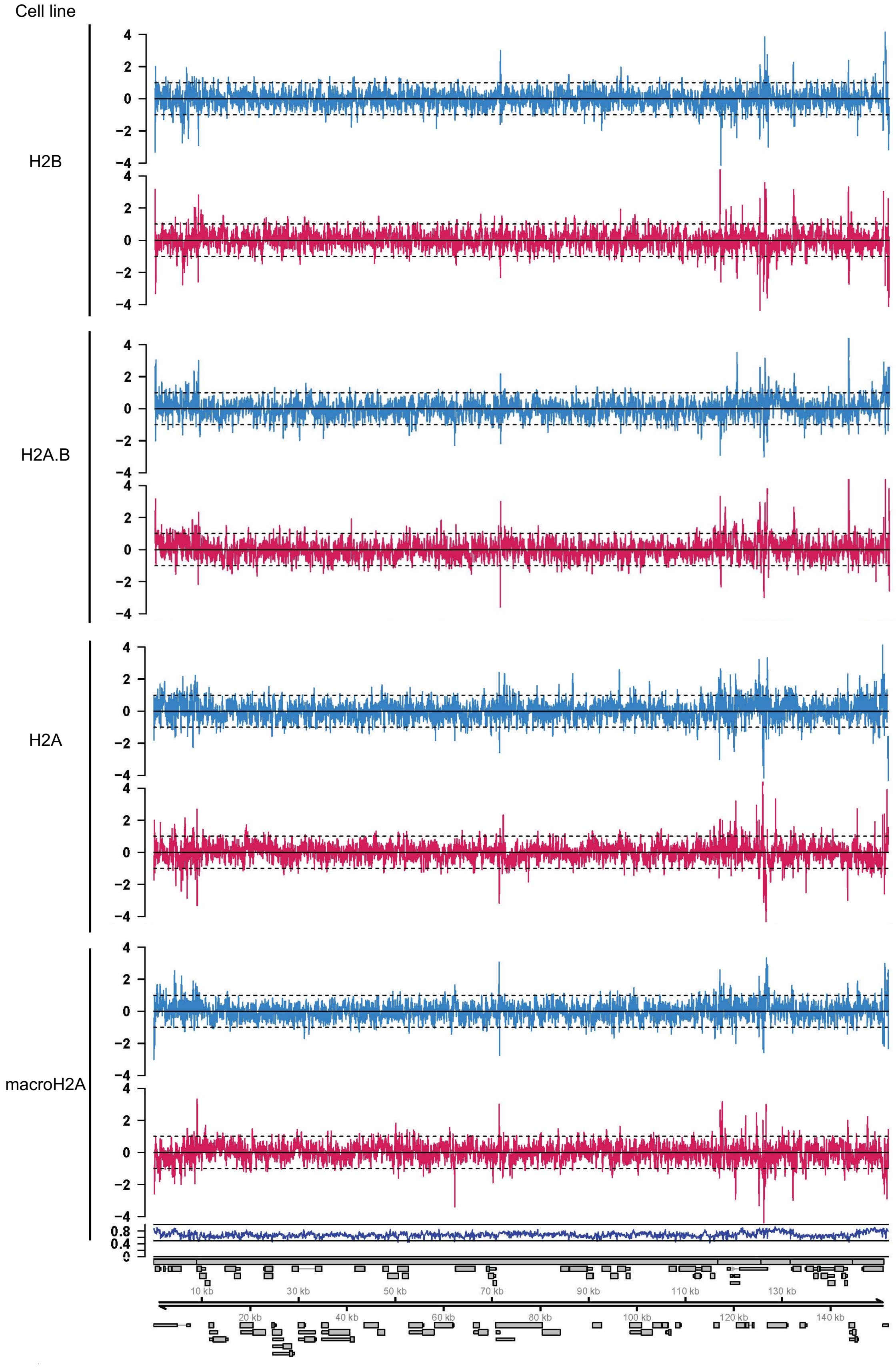

Figure S2. Flag tagged histones are equally incorporated into chromatin along the entire HSV-1 genome regardless of whether the entire genome is transcribed, or transcription is restricted to the IE loci, and no H2A variant is enriched or depleted from the IE loci when transcription is restricted to the IE genes only.

Figure S2.B

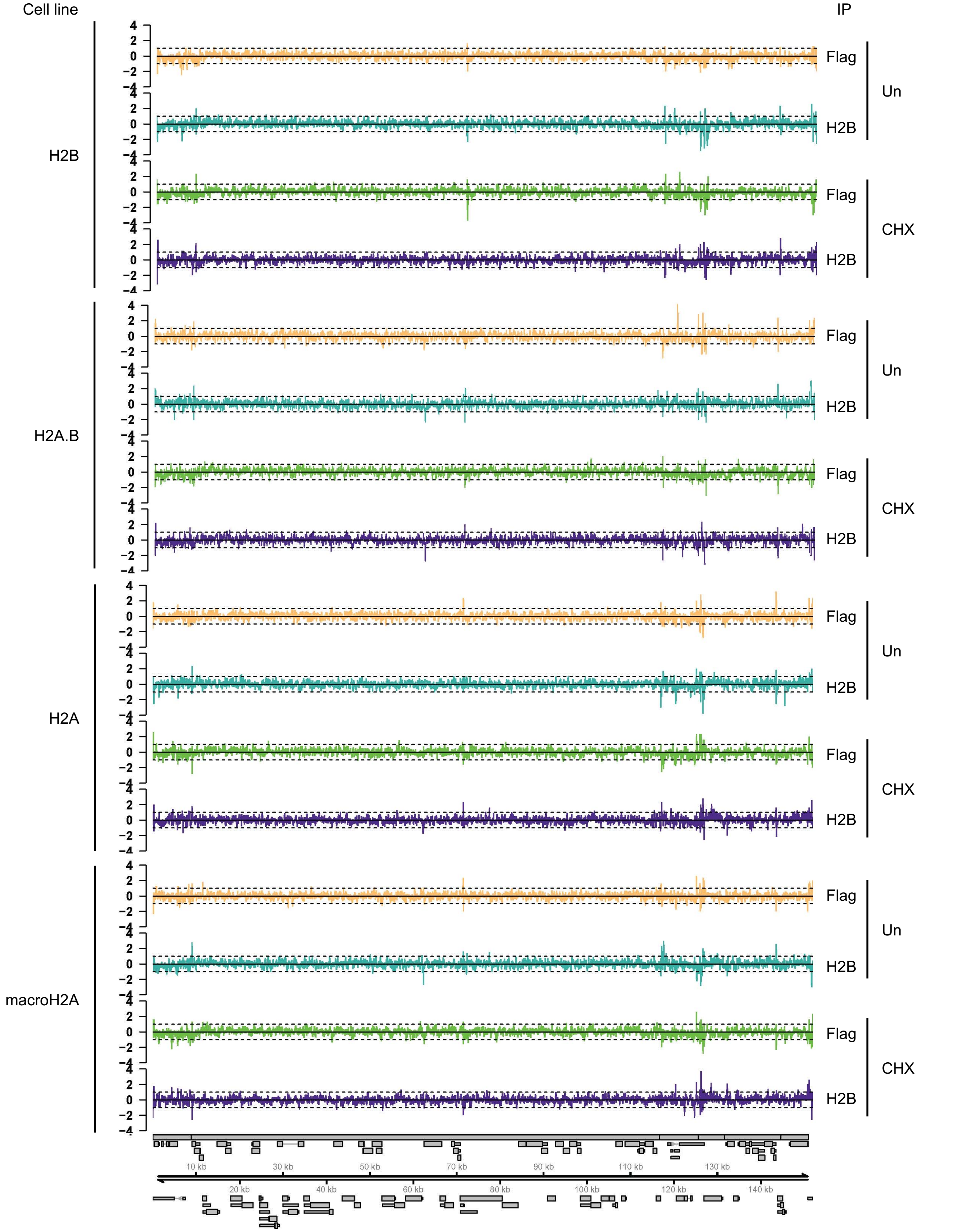

Figure S2. Flag tagged histones are equally incorporated into chromatin along the entire HSV-1 genome regardless of whether the entire genome is transcribed, or transcription is restricted to the IE loci, and no H2A variant is enriched or depleted from the IE loci when transcription is restricted to the IE genes only.
